# Anterior lumbar‒iliac fixation for unstable pelvic fractures: a novel surgical technique

**DOI:** 10.1097/JS9.0000000000002958

**Published:** 2025-08-06

**Authors:** Sheng Wang, Nan Lu, Jianlin Liu, Yongchuan Li, Lize Xiong, Aimin Chen

**Affiliations:** a Department of Traumatic Orthopedics, Shanghai Fourth People’s Hospital, School of Medicine, Tongji University, Shanghai, China; b Shanghai Key Laboratory of Anesthesiology and Brain Functional Modulation, Clinical Research Center for Anesthesiology and Perioperative Medicine, Department of Anesthesiology and Perioperative Medicine, Translational Research Institute of Brain and Brain-Like Intelligence, Shanghai Fourth People’s Hospital, School of Medicine, Tongji University, Shanghai, China

**Keywords:** incision infection, lumbar–iliac fixation, new surgical innovation, surgical approach, unstable pelvic fracture

## Abstract

The treatment of unstable pelvic fractures is a great challenge for surgeons. Traditional steel plate fixation and sacroiliac screw fixation are not sufficient to provide longitudinal support. At present, surgical lumbar‒iliac fixation is a reliable fixation technique. However, conventional lumbar‒iliac fixation surgery often involves the use of a posterior approach for surgery, which cannot observe fracture displacement and has the risk of poor surgical incision healing. In many cases, such unstable pelvic fractures also involve the fixation of iliac or pubic fractures. Under these circumstances, an additional anterior surgical incision is needed. This operation prolongs the operative time and increases the amount of bleeding. To solve the above problems, we first used a new surgical method, anterior lumbar–iliac fixation surgery, which was used to reduce and fix posterior pelvic ring unstable fractures. Iliac and pubic fractures were treated at the same time if needed. During the operation, the patient does not need to change position. Finally, the postoperative outcome of the patient was satisfactory. We hope that anterior lumbar–iliac fixation can improve the incidence of unstable pelvic fractures and reduce complications.

## Introduction

Fractures of the posterior pelvic ring are often caused by high-energy injuries, leading to instability of the pelvic ring. Early open reduction and internal fixation is a standard for treating these fractures. Surgical lumbar‒iliac fixation is a reliable fixation technique that was first proposed by Kach K in 1994^[1]^ and has been widely used to treat unstable vertical pelvic fractures. Lumbar–iliac fixation is a technique that uses the lumbar spine and pelvis as fulcrums for reduction, and it can provide longitudinal stability of the pelvis. These advantages can enable patients to perform functional exercises earlier and accelerate postoperative recovery.

However, the traditional lumbar‒iliac fixation technique requires a surgical incision in the posterior median line. Since the patient needs to maintain a supine position for a long time after the operation, the surgical incision will be under pressure, leading to drawbacks such as screw prominence irritation, surgical site infection, and poor wound healing, which will seriously affect the effectiveness of the surgery. Moreover, this surgical technique also has the problem of poor reduction during the operation because fractures cannot be observed through the incision; this greatly reduces the surgical effect^[2]^. More importantly, if a patient has an unstable pelvic fracture with breakage of the ilium, pubis, or pubic symphysis, anterior and posterior incisions are needed. The two surgical incisions also greatly increase the risk to patients during and after surgery, with obvious disadvantages in terms of operation time, blood loss, and incision infection^[3]^.

In view of the abovementioned problems, our surgical team creatively adopted the lumbar‒iliac fixation technique via the anterior approach to address these issues caused by the traditional posterior lumbar‒iliac fixation method. In this research, we preliminarily described the surgical procedures and technology and discussed the advantages of anterior surgical techniques based on a surgical case. We hereby declare that throughout the entire presentation process, no AI technology was utilized^[4]^.

## Operative technique

**Figure 1.**
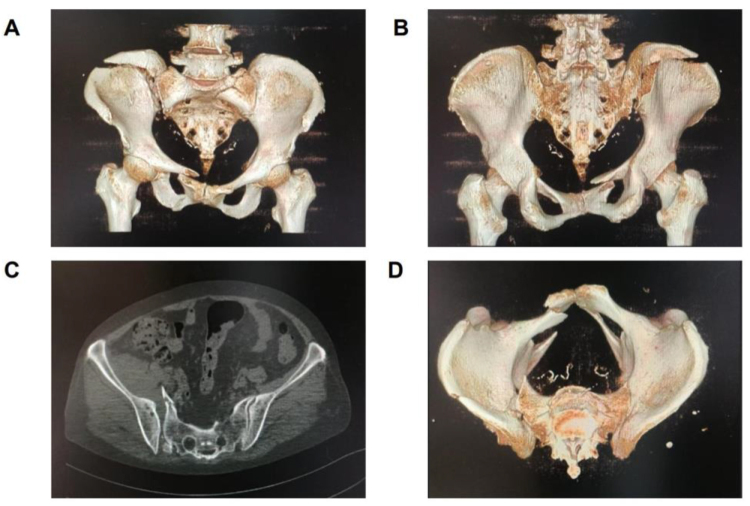
Preoperative CT results of the patient. (A) Anterior view of CT reconstruction of pelvis. (B) Posterior view of CT 3D reconstruction of pelvis. (C) CT tomography of sacroiliac joint separation. (D) Top view of CT reconstruction of pelvis.

The patient is a 31-year-old male. He was sent to our hospital for treatment after a serious car accident. The patient was transferred to a gurney, lying in a passive supine position and complaining of pain in the right hip and pelvic area. The physical examination was carried out, and the results of the pelvic compression test were positive. An orthopedic surgeon performed a CT examination and 3D reconstruction of the patient’s pelvis (Fig. 1), which suggested a fracture of the right ilium and superior pubic ramus, accompanied by a dislocation of the sacroiliac joint (crescent fracture dislocation of the pelvis). Based on the above situation, the patient was considered to receive internal fixation treatment for the fracture.

The patient was placed under general anesthesia in the supine position, and surgery was performed with a two-window iliac-inguinal incision technique (lateral window and medial window) (Supplementary Digital Content, Fig. S1, available at: http://links.lww.com/JS9/E858). First, we used the lateral window approach, and the incision length was 8–10 cm. We sharply incised and separated the abdominal muscles and iliopsoas and exposed the wing of the ilium, as well as the sacroiliac joint. We reduced the fracture of the iliac wing and fixed it with a steel plate and several screws. Then, we also reduced the shifted sacroiliac joint, and we implanted a steel plate and several screws for temporary fixation to maintain anterior and posterior stability.

**Figure 2.**
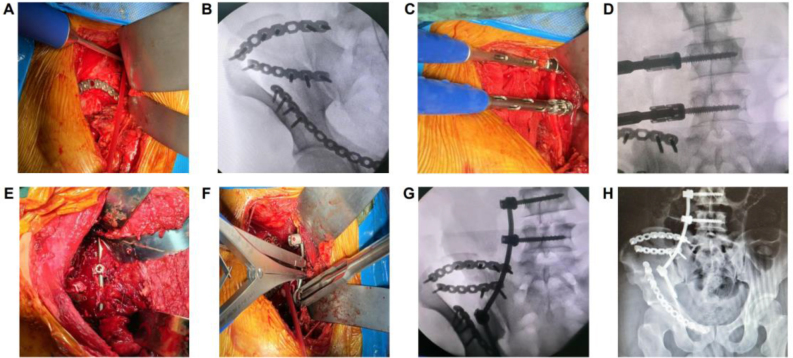
Surgical procedure (A, B). Fixation of ilium and pubis (C, D). Placement of lumbar–iliac screw (E–G). Titanium rod placement and fracture reduction (H). X-ray results of the patient’s pelvis after surgery.

Then, for exposure through a medial window, the incision is extended to the inferior border of the inguinal ligament and continues through the subcutaneous tissue. The fascia around the iliopectineal and pectineal muscles was separated to expose the superior ramus of the pubis. The tunnel beneath the muscles was established from the ilium to the superior pubic ramus. Next, the steel plate was placed through the preestablished tunnel, and the separated superior pubic ramus was reduced and fixed (Fig. 2A and B). However, the imaging results indicate that there is still significant longitudinal displacement of the sacroiliac joint.

Finally, we performed anterior lumbar‒iliac fixation. Through the lateral approach of two-window iliac-inguinal incisions, we exposed the L4.5 vertebra through limited separation of the psoas major muscle. Two screws with a diameter of 6.5 mm and a length of 5 cm were inserted through the psoas major muscle into the L4.5 vertebral body. Two screws were placed at the midpoint of the lateral side of the L4.5 vertebral body. The angle of screw placement is not exactly perpendicular to the bone surface of the vertebrae but needs to be inclined forward by 10°. During the screw insertion process, the screw should not be completely embedded in the vertebrae of L4.5, and the tail of the screw should be 0.5 cm from the bone surface of the vertebra to prevent damage to the nerve root that emerges from the posterior intervertebral foramen of L4.5 (Fig. 2C and D). Moreover, we implanted a 5 cm screw into the ilium between the inner side of the true pelvic margin and the lower edge of the sacroiliac joint in the direction of the ischial tuberosity.

After the prebent titanium rod was placed in the groove at the tail of the three screws, a spreader was used to complete longitudinal reduction and fixation. Before the spreader is used to open and reset the fracture, the screw placed in the sacrum for temporary fixation should be loosened; after the spreading process is complete, the screw should be retightened (Fig. 2E–G). All critical steps during the operation were performed under fluoroscopy with a C-arm base.

## Results

**Figure 3.**
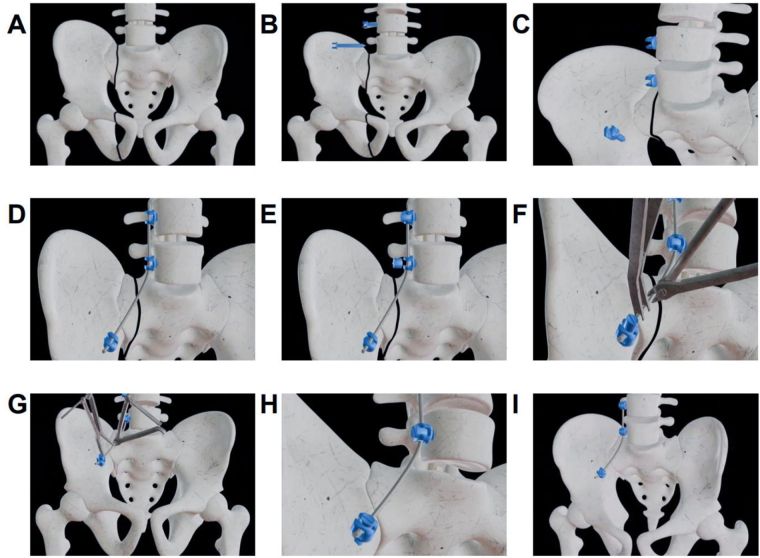
Virtual image of anterior lumbar–iliac fixation for unstable pelvic fractures. (A) Pelvic fracture with displacement. (B) Screws inserted at the L4 and L5 level. (C) Screws inserted at the iliac bone. (D) Titanium rod inserted. (E) Tail cap of the screw. (F) Pelvic reduction using a reduction clamp. (G) Reduction completed. (H) Placement of the screw tail cap. (I) Internal fixation surgery completed.

The patient was given intravenous antibiotics before the operation. The drainage tube was removed 48 h after the operation. The incision healed well. After the operation, the patient underwent a pelvic plain film examination, and the results showed that the effects of fracture reduction and internal fixation were excellent, as shown in Figure 3H. The pelvic Matta score was then calculated, and the separation and displacement of the posterior pelvic ring could not be observed (anatomical reduction). and the rating is optimal. At approximately 6 months after surgery, the function of the pelvis after surgery was evaluated by the Majeed Pelvic Outcome Score, with a score of 30 (of 30) for pain, 16 (of 20) for work, 10 (of 0) for sitting, 4 (of 4) for sexual function, and 32 (of 36) for standing, resulting in a total score of 92 (of 100). The evaluation grade was excellent.

## Discussion

Unstable pelvic fractures are often caused by high-energy injuries, and the treatment of unstable pelvic fractures is a great challenge for trauma surgeons. Our group has creatively proposed the anterior lumbar‒iliac fixation technique based on years of clinical experience. The whole operation process is shown in Figure 3. Here, the new anterior lumbar‒iliac fixation technique is compared with various original relevant surgical techniques.

The sacroiliac screw technique and plate screw fixation technique are often used for unstable fractures of the posterior pelvic ring. The sacroiliac screw surgery was first proposed by American Matta J in 1989^[5]^. In 1993, M J Albert from the United States proposed the technique of plate fixation for pelvic fractures^[6]^. After the two techniques were proposed, they developed rapidly and were applied to clinical patients. However, during more than 30 years of clinical application, two techniques have revealed some problems, and research from Italy has shown that both techniques have insufficient longitudinal stability^[7]^, which is particularly prominent in steel plate fixation surgeries^[8]^. With respect to the use of the sacroiliac screw fixation technique, a study from England reported that surgery requires a good level of experience and preoperative preparation; otherwise, the incidence of screw malpositioning is high, and neural damage can also occur^[9]^.

Lumbar–iliac fixation is a reliable longitudinal fixation technique and was first proposed by Kach K in 1994^[1]^. After posterior lumbar–iliac fixation, the longitudinal stability of unstable pelvic fractures has improved, and nerve relaxation can also be achieved. However, this technique also has disadvantages. Studies from Finland have confirmed that the technique is prone to causing damage to nerves, which emanate from the intervertebral foramen of the lumbar vertebrae^[2]^. Research from Germany confirmed that broken bone fragments cannot be observed through the posterior approach and that posterior lumbar pelvic fixation has a greater probability of causing screw prominence irritation, surgical site infection, and wound cleft because of the long-term pressure on the incision due to the supine position^[10]^. These problems can all be solved by the anterior incision in our new technology. In cases of multiple pelvic fractures, if a patient has an unstable pelvic fracture with breakage of the ilium, pubis, or pubic symphysis, surgery requires anterior and posterior incisions experientially. However, this method requires changing the patient’s position, increasing the risk of anesthesia, blood loss, and operation time^[3]^. Our new surgical approach can effectively reduce the aforementioned complications.

Through this research, we propose anterior lumbar pelvic fixation surgery for the first time in the world. The manuscript elaborates on the advantages of the operation and suggests that in the future, this surgical method can be used to solve more clinical problems.

## Conclusion

We are encouraged by the results of this research, in which we creatively proposed an approach to anterior lumbar‒iliac fixation. In conclusion, this technique provides good reduction and longitudinal stability, shortens the operative time, and reduces surgical complications. This technique may be a milestone in the treatment of unstable pelvic fractures, and more clinical research is needed in the future.

## Data Availability

Data are not from the database. All data can be found in the supplementary material.
